# Potential of Exercise for Prevention of Cardiovascular Disease in Survivors of Childhood Hodgkin Lymphoma

**DOI:** 10.1016/j.jaccao.2025.11.002

**Published:** 2026-02-17

**Authors:** Aron Onerup, Qi Liu, Shizue Izumi, José Miguel Martínez-Martínez, Stephanie B. Dixon, Eric J. Chow, Melissa M. Hudson, Claire Snyder, Paul C. Nathan, Gregory T. Armstrong, Kirsten K. Ness, Yutaka Yasui

**Affiliations:** a Department of Epidemiology and Cancer Control, St. Jude Children’s Research Hospital, Memphis, Tennessee, USA; b Department of Pediatrics, Institute of Clinical Sciences, Sahlgrenska Academy, University of Gothenburg, Gothenburg, Sweden; c School of Public Health, University of Alberta, Edmonton, Alberta, Canada; d Faculty of Data Science, Shiga University, Hikone, Japan; e Department of Oncology, St. Jude Children’s Research Hospital, Memphis, Tennessee, USA; f Fred Hutchinson Cancer Center, Seattle, Washington, USA; g Departments of Medicine, Oncology, and Health Policy and Management, Johns Hopkins Schools of Medicine and Public Health, Baltimore, Maryland, USA; h Division of Hematology and Oncology, The Hospital for Sick Children, University of Toronto, Toronto, Ontario, Canada; i Department of Biostatistics, St. Jude Children’s Research Hospital, Memphis, Tennessee, USA

**Keywords:** cardio-oncology, exercise, exercise oncology, heart failure, Hodgkin lymphoma

**Figure:**
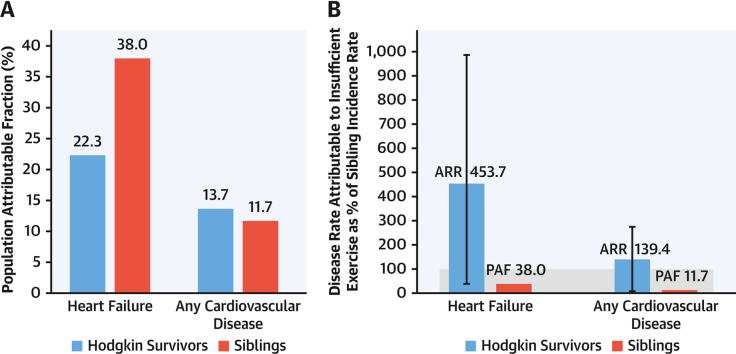

Survivors of childhood Hodgkin lymphoma (HL) are at increased risk for cardiovascular disease (CVD) from treatment exposures. Although it has been reported that exercise is associated with lower CVD risk in survivors of HL,^1^ the potential impact of exercise on CVD in the HL survivor population compared with the general population is not known.

The potential impact of a risk factor on disease incidence in a population can be estimated using the population attributable fraction (PAF), which estimates the proportion of the disease burden in a population that would be prevented if the risk factor was not present,^2^ reflecting both the effect size and prevalence of a risk factor. Clinical populations share risk factors for disease with the general population. For example, insufficient physical activity is a risk factor for CVD both in the general population and in survivors of HL.^1^^,^^3^ It is possible that shared risk factors interact with population-specific risk factors (eg, cancer treatment), amplifying the importance of the risk factor in the clinical population. Because of differences in disease incidence, and potentially also in the prevalence of the risk factor, direct comparisons of PAFs between a clinical population of interest and the general population do not quantify the potential impact of a risk factor on disease burden in a comparable fashion between the 2 populations.

The aim of this study was to estimate the potential impact of not meeting exercise recommendations on CVD incidence in HL survivors and compare it with the potential impact in sibling control subjects. Given that there are no established methods for comparing the potential impact of a risk factor between populations that incorporate differences in both disease incidence and risk factor prevalence, we propose the attributable relative rate (ARR), an estimate that enumerates the reduction in the incidence rate of disease in a population of interest (survivors of HL) by removing a risk factor of interest, relative to the incidence rate of disease in the reference population (sibling control subjects).

To estimate the ARR, the PAF in the population of interest was first estimated: the PAF of a CVD outcome in HL survivors in a scenario in which survivors currently not meeting exercise recommendations all met the recommendations.^2^ Then, the PAF was multiplied by the relative rate (RR) estimate for the outcome in the population of interest compared with the reference population, resulting in the ARR. A 95% CI around the ARR was obtained through nonparametric bootstrap with 10,000 iterations.

We used data from the CCSS (Childhood Cancer Survivor Study), a multi-institutional cohort with longitudinal prospective follow-up of 5-year survivors of childhood cancer,^4^ to examine not meeting exercise recommendations and the relationship with CVD among 2,357 HL survivors and 3,949 sibling comparators. Self-reported vigorous exercise was transformed into MET hours per week, and all possible MET hours per week values were categorized as <3.0 MET-h/wk, 3.0 to 8.9 MET-h/wk, or meeting exercise recommendations (≥9.0 MET-h/wk). The CCSS was approved by the Institutional Review Board at each institution. Piecewise exponential models estimated the RRs for >3 and 3.0 to 8.9 MET-h/wk and PAFs of not meeting exercise recommendations for new-onset CVD, adjusting for age, sex, race/ethnicity, and treatment exposures (survivors only). CVD incidence rates attributable to not meeting exercise recommendations between HL survivors and siblings were compared, presented as ARRs.

Among HL survivors, 2,068 (87.7%) were exposed to chest radiation and 252 (10.7%) to high-dose (≥250 mg/m^2^) anthracyclines. Survivors of HL had a considerably higher rate of heart failure (RR: 20.3; 95% CI: 12.6-32.6) and any CVD (RR: 10.2; 95% CI: 8.2-12.6) during follow-up, compared with sibling control subjects.

For the associations between vigorous exercise and CVD, survivors who reported no vigorous exercise (<3.0 MET-h/wk) had higher rates of subsequent heart failure (RR: 1.6; 95% CI: 1.1-2.3) and any CVD event (RR: 1.3; 95% CI: 1.1-1.7) than those meeting exercise recommendations (≥9.0 MET-h/wk). This corresponded to PAF estimates of 22.3% (95% CI: 1.9%-40.8%) and 13.7% (95% CI: 0.7%-25.8%) for heart failure and any CVD event, respectively, for not meeting exercise recommendations. In siblings, the corresponding PAFs were 38.0% (95% CI: 0%-83.6%) and 11.7% (95% CI: 0%-38.3%), respectively (Figure 1A).

**Figure 1:**
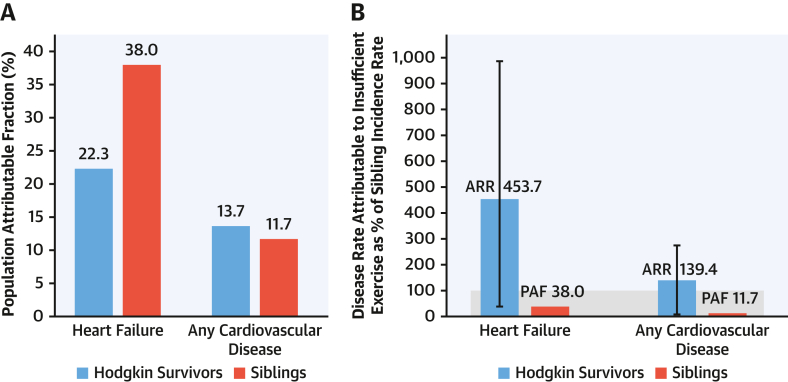
Population Attributable Fractions and Attributable Relative Rates for Not Meeting Exercise Recommendations in Survivors and Siblings (A) Population attributable fractions (PAFs) for not meeting exercise recommendations for heart failure and any cardiovascular disease in survivors and siblings. (B) Attributable relative rates (ARRs) for not meeting exercise recommendations for heart failure and any cardiovascular disease in survivors, compared with PAFs and the total incidence rates for siblings. Incident rate of siblings as 100%. The figure illustrates the benefit of using the ARR for comparison of the potential impact of a shared risk factor (insufficient exercise) on an outcome (cardiovascular disease) between 2 populations (survivors and siblings).

The ARR for not meeting exercise recommendations for heart failure in HL survivors was 453.7% (95% CI: 41.7%-986.9%) of the disease rate in the sibling control subjects, corresponding to 11.9 times the magnitude observed in sibling control subjects (PAF = 38.0%) (Figure 1B). Although the PAFs in HL survivors (22.3%) and siblings (38.0%) might suggest a higher relative burden among siblings, these values do not account for differences in baseline disease incidence and exposure prevalence between the 2 groups. The ARR helps clarify this comparison by showing the absolute impact of the risk factor across populations. In this case, it indicates that the 22.3% of heart failure in HL survivors attributable to not meeting exercise recommendations corresponds to 453.7% of the disease rate in siblings, emphasizing the much greater absolute burden among survivors despite their lower PAF. For any CVD event, the ARR (139.4%; 95% CI: 7.2%-273.2%) also indicated that the magnitude of the CVD incidence attributable to not meeting exercise recommendations in HL survivors was approximately 12 times larger than the magnitude in sibling control subjects (11.7%) (Figure 1B).

In summary, our findings using the ARR suggest that the disease rates attributable to not meeting exercise recommendations in survivors are equivalent to 1.4 times the total rate of any CVD (ARR: 139.4%) and 4.5 times the total rate of heart failure (ARR: 453.7%) in sibling control subjects. Although the PAFs in survivors and siblings suggest a larger relative burden in siblings, these findings emphasize that the absolute burden of disease attributable to inadequate exercise is far greater in survivors. These results offer a modifiable target and provide rationale for exercise interventions aimed at reducing CVD in survivors at high risk. Beyond this specific context, this study also serves as an illustrative example that the ARR can be a useful tool to measure the potential impact of a risk factor on the rate of a disease in clinical populations at increased risk for a disease of interest, using a reference rate as the unit of measurement. Examples of this could be the potential impact of insufficient physical activity or hypertension on CVD in diabetes populations or insufficient activity or obesity on CVD in populations with hypertension.

## Funding Support and Author Disclosures

This work was supported by the National Cancer Institute (grant CA55727, Gregory T. Armstrong, principal investigator). Support to St. Jude Children’s Research Hospital was also provided by the Cancer Center Support grant (CA21765, C. Roberts, principal investigator) and the American Lebanese-Syrian Associated Charities. Dr Onerup was supported by grants from the Swedish Research Council (2022-00166 and 2024-02859), the Swedish Childhood Cancer Fund (PD2023-0003), and the Gothenburg Medical Society (GLS-999210). The funders had no role in study design; in the collection, analysis, or interpretation of data; or in the decision to submit the paper for publication. The authors have reported that they have no relationships relevant to the contents of this paper to disclose.
